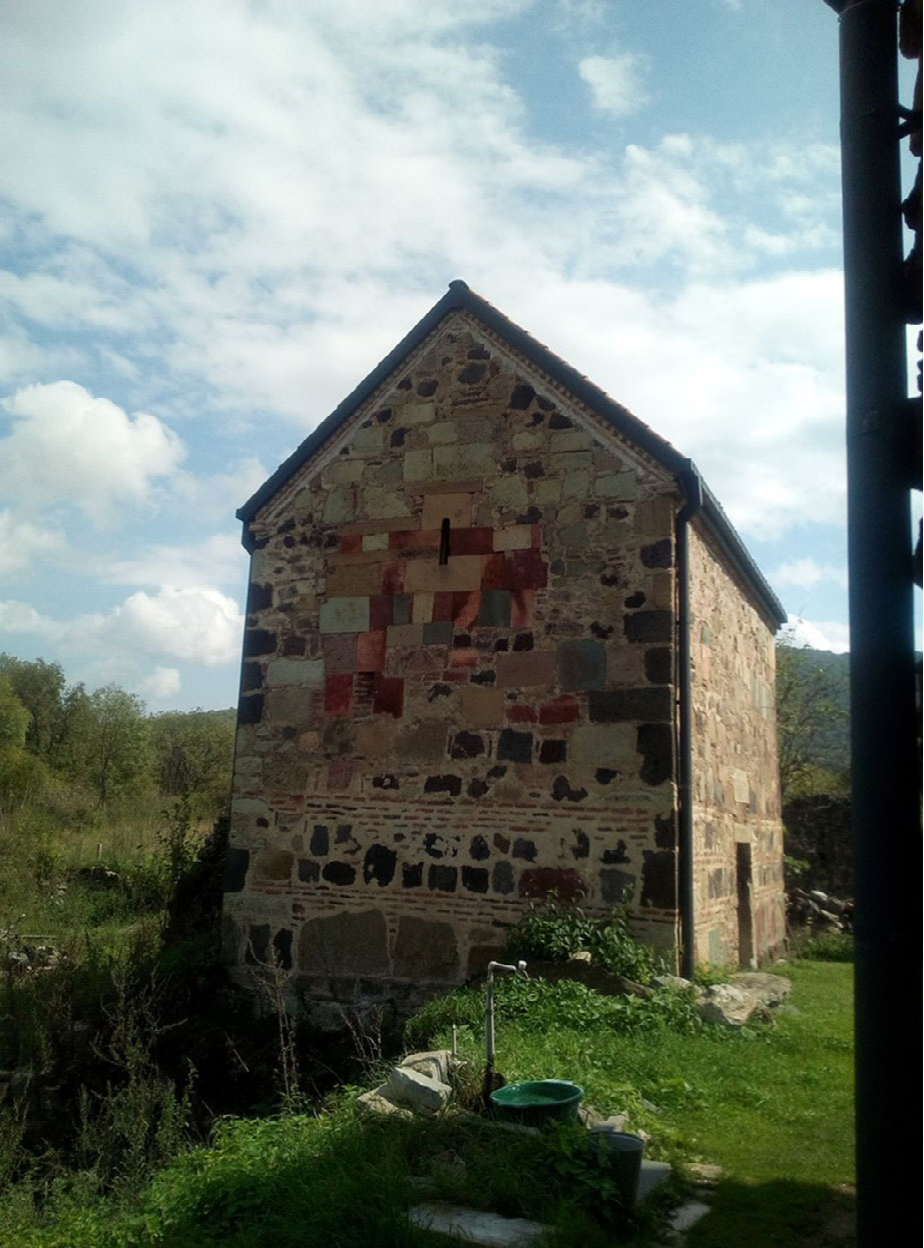# Bioarchaeology: a profitable dialogue between microbiology and archaeology

**DOI:** 10.1111/1751-7915.13527

**Published:** 2020-02-13

**Authors:** Harald Brüssow

**Affiliations:** ^1^ KU Leuven Leuven Belgium

## Abstract

The cultivation of yeasts from up to 5000‐year‐old beer vessels in Israel allows insights into early domestication of microbes for food production, but also raises questions about long‐term survival of microbes under dormancy or slow growth.

The use of physical analytical methods has transformed archaeology. A milestone was the introduction of absolute dating methods in the 1950s based on the determination of the radioactive decay from the carbon‐14 isotope in organic material discovered at the excavation site. As an illustration, the Cortaillod culture in a lake region from Western Switzerland was dated by this approach to 3000 to 2600 BC. Thanks to an exceptional preservation of artifacts in a humid soil, this culture has yielded numerous archaeological findings documenting early agricultural practices in prehistoric Switzerland. The discovery of pitchers with many holes in the bottom of the vessel suggested to some archaeologists ‘cheese strainers’ and thus early cheese making activity in Switzerland. Other archaeologists disagreed and proposed filtration of fruit juice as alternative use. By archaeological means, it was difficult to settle the dispute. In the meantime, rock paintings from the Sahara were discovered that documented even earlier dairy activities. While it is clear that these scenes stem from a greener past of the Sahara, rock paintings are difficult to date. Here the chemical analysis of milk fat residues extracted from the potsherds and the investigation of the stable carbon‐13 isotope distribution allowed a dating of this Saharan dairy activity into the fifth millennium BC. The development of the chemical methodology for milk fat analysis pioneered by R.P. Evershed from the Bristol Biogeochemistry Research Center substantially enriched the toolbox of archaeologists. The systematic investigation of pottery vessels derived from archaeological sites covering the Near East to South‐Eastern Europe demonstrated that dairy activity started in the seventh millennium BC in the north‐western Turkey and spread from there to Europe. Vessels with holes in the bottom associated with milk fat residues documented cheese making activity in Poland at the sixth millennium BC (Salque *et al.*, 2013). Swiss cheese making could thus well have a 5000‐year‐old tradition. Biology, particularly genetics and DNA sequencing, has made important contributions to archaeological research into the origin of agriculture by documenting the domestication of animals and plants going back to the Neolithic Revolution. Microbiologists have argued that compared with research into the domestication of plants and animals, the domestication of microbial agents involved in food fermentation (barley‐beer, vine‐wine, ruminant milk‐cheese) has been neglected while fermentation might have played an important role in early agrarian societies.

This situation is, however, about to change. A recent paper published in *mBio* combines microbiology with archaeology (Aoizerat *et al.*, 2019). Israeli scientists led by M. Klutstein and R. Hazan from the Hebrew University of Jerusalem selected ceramic vessels that were by archaeologists classified as storage vessels for alcoholic beverages. This diagnosis was further supported by the detection of characteristic chemical traces indicative of alcoholic fermentation. The vessels dated from three different historical periods. The oldest were of Egyptian origin and belonged into the Bronze Age (ca.3000 BC), the next corresponded to the Philistine Iron Age (ca. 850 BC) and the youngest were from the Persian period (ca. 4th century BC). By scanning electron microscopy they associated yeast‐like structures with the clay material. When they incubated the ceramic material first on plates that excluded bacterial growth and then re‐plated the colonies on non‐selective media, they isolated typical yeast colonies and visualized yeast cells by microscopy. Subsequent whole genome sequencing attributed two ‘Egyptian’ isolates to *Saccharomyces delphensis,* known to colonize African figs. Another Egyptian potsherd isolate corresponded to a yeast known to contaminate Nigerian beer. The Philistine vessels yielded two yeast strains: one was attributed to a yeast used in African beer brewed with sorghum malt. The other was a close relative to *Saccharomyces cerevisiae* used nowadays in wine making. The Persian vessel yielded a yeast known from an Ethiopian honey beverage and the chemical analysis indeed suggested a mead storage vessel. In an experimental archaeology approach, several of the isolates produced on malt wort a drinkable beer. Can we now drink 5000‐year‐old Egyptian beer? An answer depends on the contamination controls conducted by the authors. The investigators isolated 6 strains from 21 vessels that were typical containers for fermented beverages. In comparison, they isolated only two strains from 110 controls that comprised ceramics not known to contain beverages and sediments surrounding the findings. Only two yeast colonies were isolated from them: one from an oil lamp and one from a stone. The stone isolate was *Candida albicans*, a pathogenic yeast and a likely contemporary contaminant, while the oil lamp isolate resembled mead‐associated yeasts. Since these yeasts are also natural members of the microbiome of olive oil, their association with oil lamps is easily explained. They might be the literal exception, which confirms the rule. Overall, the evidence seems quite good that ancient yeast was isolated from at least some of these archaeological ceramic samples.

Important questions arise from this report. One is how far back in time can we isolate ‘ancient’ microbes? The Israeli scientists argue that occasional supply of moisture and nutrients might have allowed the ceramic‐associated yeasts to grow and to survive over millennia. They suggested that bacteria, which are tougher than yeasts, and particularly bacterial spores, which are designed by evolution as the hardiest survival form of bacteria, might yield viable isolates from even older materials. Claims to the reactivation of ancient *Bacillus* spores have indeed been made. R. Vreeland and colleagues reported in a *Nature* paper from 2000 the isolation of a halotolerant *Bacillus* isolate from brine inclusions in salt crystals, which are 250 million‐year‐old. The crystals showed no evidence of damage or secondary alteration, and the crystals were washed with strong acid and alkali before recovering the brine fluid. Such purported longevity of spores are interesting in the context of the panspermia hypothesis according to which life on earth might have been seeded from extraterrestrial space. Microbiologists remained skeptical. Are spores able to survive for so extended periods of time? Spore survival from the time of L. Pasteur has been documented. That spores can survive for millennia is commonly anticipated, but maintaining intact biological structures for 250 million years in a closed 10 microlitre brine space without energy input seems to hurt thermodynamic principles. Furthermore, no spore structures were visualized in the brine and finally, the 16S rRNA gene sequence was 99 % identical with contemporary *Bacillus marismortui*. Such a degree of sequence conservation seems at odds with ideas about the pace of bacterial evolution. However, this Nature report is in line with a *Science* paper from R. Cano and M. Borucki published in 1995 where they describe a *Bacillus sphaericus* spore activation from the gut of bees trapped in 25‐ to 40‐million‐year‐old amber. This case is better documented since spore‐like structures were documented in the abdomen of these bees. In addition, *Bacillus* DNA was extracted from the abdomen of the entrapped bees and the amber was washed before extraction with a mixture of chemicals (glutaraldehyde, bleach, ethanol) efficiently attacking biological structures. However, with respect to the rRNA gene sequence, the isolates were close relatives of extant *B. sphaericus* raising again doubts about their antiquity. Current *Bacillus* spore survival experiments explore more modest time intervals like half of a millennium. They are inspired by experiments with permafrost sediments. In a PNAS paper from 2007, E. Willerslev and colleagues used a long, but conserved 4‐kbp amplification product around rRNA genes as a proxy for intact DNA and a liberal criterion for potential viability. In the 5 to 30 kilo‐year (Kyr) age range, spore‐forming bacteria accumulated hydrolytic damage largely preventing PCR amplification of such fragments. In 400 to 600 Kyr‐old ice cores, no spore formers gave amplification products, demonstrating that dormancy, which confers high resistance against harsh environmental conditions, is not a long term survival strategy. These authors still detected 4‐kb amplification products in non‐spore forming Actinobacteria and linked this long‐term survival activity to DNA repair mechanisms in cells displaying still a very low basic metabolic activity not seen in dormancy. An *AEM* paper from 2019 by R. Liang suggested viable microorganism in up to 1 million‐year‐old permafrost soil on the basis of lower than expected amino acid racemization. The oldest layers were enriched in spore‐forming Firmicutes. In contrast, a recent *mBio* report using metagenome, meta‐transcriptome and metabolome analyses in up to 50 000‐year‐old deep marine sediments revealed fascinating mechanisms of long‐term microbial survival in energy‐limited environments (Bird *et al.*, 2019). The investigated unculturable microbes showed trehalose production, which stabilizes proteins and nucleic acids, and biochemical pathways that exploit scarce substrates, but included also unusual mechanisms like the counter‐intuitive export of amino acids. The latter was interpreted as a mean to slow down own cell growth. Since competition is not well possible in resource‐limited environments (nobody has enough energy to outcompete anybody), cross‐feeding between extremely slow‐growing microbes might be a better strategy than competition to allow survival in deep sediments. In contrast to Bacilli that accidentally got trapped and purportedly survived in the salt and amber inclusions, the deep marine sediments might represent the biggest biomass on earth and the extremely slow microbial growth might be the compromise between longevity and thermodynamics.

It thus seems plausible to describe a viability limit for ancient organisms by the absence of growth and repair to the chemical stability of the genetic material. Since RNA is chemically more labile than DNA, the oldest sequenced RNA virus genome is a 1000‐year‐old maize virus. Notably, it belonged to a virus group with double‐stranded RNA genome making it chemically more resistant than single‐stranded RNA. DNA is chemically more resistant than RNA, but unrepaired DNA will be fragmented into 100 bp pieces or heavily crosslinked in < 1 million years under optimal storage conditions. This estimate is corroborated by the currently oldest ancient DNA sequencing record held by M. Meyer, S. Pääbo and colleagues with heavily damaged 430 000‐year‐old hominin DNA from Spain.

While these data make the isolation of more than 1 million‐year‐old microorganisms unlikely, they do not exclude the yeast isolation results of the Israeli researchers. As everywhere in science, independent confirmation by other laboratories will be essential to separate facts from artifacts. Anticipating that the yeast data from the archaeological samples of Israel are correct, it might be interesting to extend these microbial cultivation experiments to oldest ceramics with chemical traces of wine making in the form of tartaric acid, currently held by pots of the Shulaveri culture in the Republic of Georgia, which is dated to 6000 to 5000 BC, as reported by D. Lordkipanidze and colleagues in a 2017 *PNAS* paper. The oldest ceramic vessels with chemical traces of dairy fermentation like the Polish cheese strainers might be another fascinating target for microbial isolation. Such isolates could provide insights into the domestication of microbes during the early stages of agriculture, food processing and food preservation in prehistoric times.

At the end of this short overview on bioarchaeology, one should mention a new technology that might help where DNA sequencing reaches its current technological limits. Georgian archaeologists discovered a *Homo erectus* specimen in Dmanisi/ Georgia dated to 1.8 million years ago. An slightly older *Homo erectus* specimen was even found in China, demonstrating an out‐of‐Africa movement long before *Homo sapiens* followed suit. No DNA suitable for sequencing could be recovered from the *H. erectus* specimen. However, proteins are more stable than DNA; collagen had been be recovered from bones of this age. However, collagen is a protein with a highly repetitive primary structure making it of limited use for establishing ancestral relationships between skeletons too old for DNA sequencing. D. Lordkipanidze, E. Willerslev and colleagues argued that teeth are the most resistant tissue in the human body and also the most abundant archaeological findings. These researchers found in 1.8 million‐year‐old teeth from Dmanisi about 1000 peptide matches to the dental enamel proteome, which showed chemical modifications proving their antiquity (Cappellini *et al.*, 2019). The *H. erectus* teeth were too valuable to be used for this proof of principle work, which was done with animal teeth allowing the molecular reconstruction of phylogenetic relationships of Pleistocene rhinoceros. Forty thousand‐year‐old teeth from Neanderthals have also revealed substantial insights for microbiologists (Weyrich *et al.*, 2017). L. Weyrich, A. Cooper and colleagues sequenced DNA recovered from dental calculus of these Neanderthals. With that data set they did not only distinguish meat‐eating Neanderthals in Belgium from vegetarian Neanderthals in Spain, but could also define an oral microbiome of Neanderthals distinct from that of modern humans (with more Actinobacteria and less Bacteroidetes). In the dental calculus, they could identify caries‐ and periodontitis‐associated bacterial pathogens as well as a gastroenteritis pathogen. An Archaea *Methanobrevibacter oralis* genome was sequenced and is currently the oldest microbial draft genome. It diverged from the corresponding species associated with modern humans more than 100 000 years ago. One individual had a dental abscess that was possibly treated with aspirin produced by a poplar extract and an antibiotic produced by a food contaminating *Penicillium* species. Research papers like this document that the relationship between archaeology and microbiology becomes increasingly an asset for both disciplines.

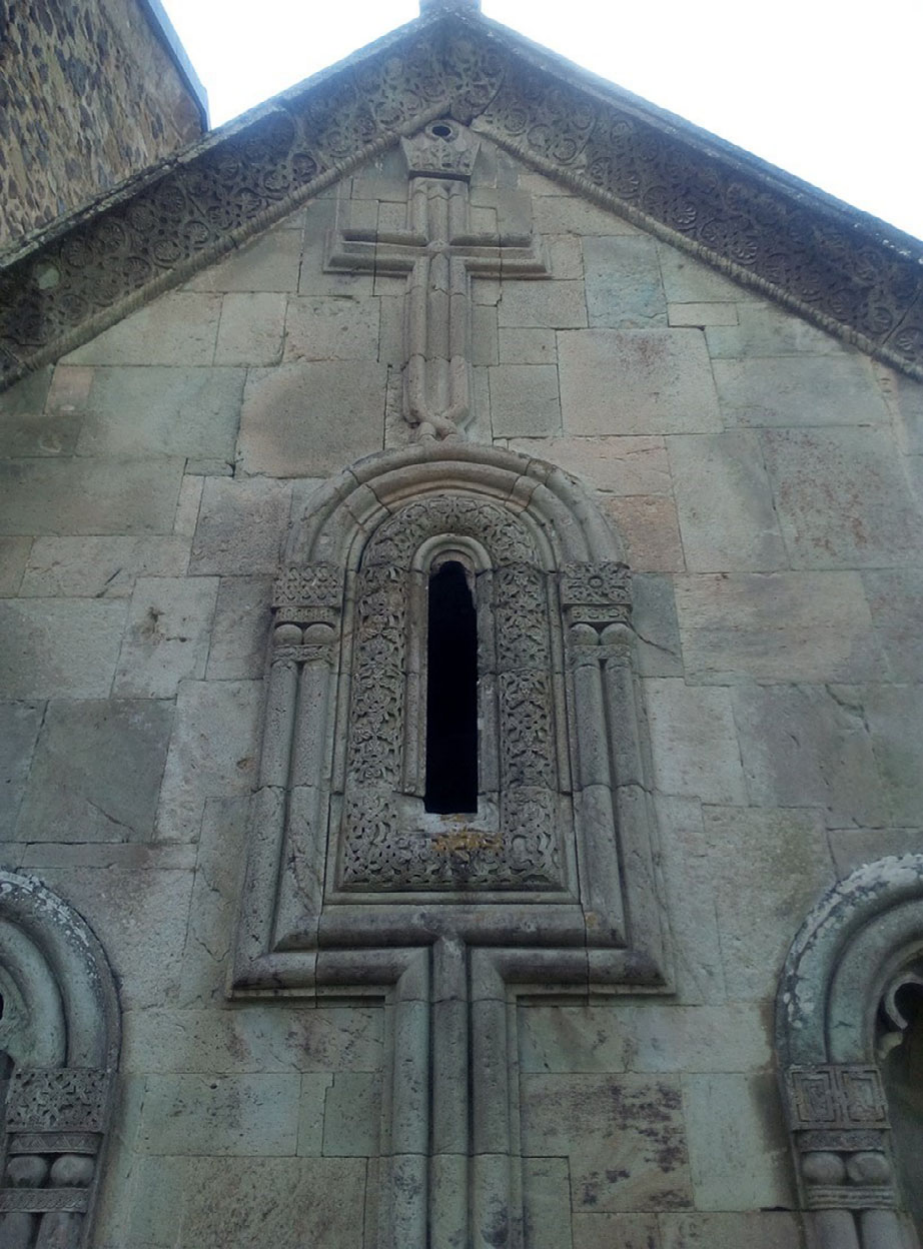

Islamic/ Christian co‐culture in Dmanisi/Georgia, near the site where a 1.8 million‐year‐old *Homo erectus* was discovered.